# Genetic Parameters for Tolerance to Heat Stress in Crossbred Swine Carcass Traits

**DOI:** 10.3389/fgene.2020.612815

**Published:** 2021-02-04

**Authors:** Maria Usala, Nicolò Pietro Paolo Macciotta, Matteo Bergamaschi, Christian Maltecca, Justin Fix, Clint Schwab, Caleb Shull, Francesco Tiezzi

**Affiliations:** ^1^Department of Agricultural Science, University of Sassari, Sassari, Italy; ^2^Department of Animal Science, North Carolina State University, Raleigh, NC, United States; ^3^Acuity Ag Solutions, LLC, Carlyle, IL, United States; ^4^The Maschhoffs, LLC, Carlyle, IL, United States

**Keywords:** heat stress, fat and muscle growth, genotype by environment interaction, heritability, single-step genomic BLUP

## Abstract

Data for loin and backfat depth, as well as carcass growth of 126,051 three-way crossbred pigs raised between 2015 and 2019, were combined with climate records of air temperature, relative humidity, and temperature–humidity index. Environmental covariates with the largest impact on the studied traits were incorporated in a random regression model that also included genomic information. Genetic control of tolerance to heat stress and the presence of genotype by environment interaction were detected. Its magnitude was more substantial for loin depth and carcass growth, but all the traits studied showed a different impact of heat stress and different magnitude of genotype by environment interaction. For backfat depth, heritability was larger under comfortable conditions (no heat stress), as compared to heat stress conditions. Genetic correlations between extreme values of environmental conditions were lower (∼0.5 to negative) for growth and loin depth. Based on the solutions obtained from the model, sires were ranked on their breeding value for general performance and tolerance to heat stress. Antagonism between overall performance and tolerance to heat stress was moderate. Still, the models tested can provide valuable information to identify genetic material that is resilient and can perform equally when environmental conditions change. Overall, the results obtained from this study suggest the existence of genotype by environment interaction for carcass traits, as a possible genetic contributor to heat tolerance in swine.

## Introduction

The increased relevance of heat stress to livestock industries is due to concerns in animal welfare as well as its economic impact. Estimates of annual financial loss to the pork industry that are attributable to heat stress range between $299 and $316 million. Of these losses, those for growing–finishing pigs are estimated to be $202 million (St-Pierre et al., 2003).

The biological mechanism by which heat stress impacts production and reproduction in pigs has been widely documented by different authors (Pearce et al., 2013; Johnson et al., 2015; Sanz Fernandez et al., 2015). However, very little is known about the detrimental effects of heat stress on carcass and meat quality traits in pigs.

Carcass characteristics are crucial for the profitability of pork producers because the carcass price is often adjusted based on these components, particularly backfat (with negative emphasis) and loin depth (with positive emphasis). The knowledge of the extent of genotype by environment interactions for these traits and the ability to identify pigs that are less susceptible to heat stress would greatly increase the competitiveness and efficiency of the pork industry in the face of climatic changes.

Currently, selection tools for improving heat tolerance or adaptability are not implemented in swine genetic evaluations. Many studies explored the genetic variation in heat tolerance in several livestock species using reaction norm models (Ravagnolo et al., 2000; Zumbach et al., 2008; Carabaño et al., 2016; Tiezzi et al., 2017), suggesting the existence of a genetic determinism of heat tolerance. Reaction norm models are often implemented as random regression models. The use of these models allows modeling the effect of a genotype as a function of environmental conditions through the estimation of genetic parameters over the range of an environment-dependent covariate (Legarra et al., 2009; Santana et al., 2016).

Most of the studies regarding the G × E interaction performed in swine populations have been focused on live body weight and growth performance (Merks, 1986; Bidanel and Ducos, 1996; Godinho et al., 2018, 2019) or carcass weight (Zumbach et al., 2008; Fragomeni et al., 2016a,b).

Studies on the carcass characteristics of the pig are not available, despite of their economic importance. Therefore, the objective of this study was to estimate genetic parameters for heat tolerance leveraging on a potential genotype × environment interaction for carcass quality traits of commercial crossbred pigs.

## Materials and Methods

### Animal Data

Animal use approval was not needed for this study because the data were from an existent database and were provided by The Maschhoffs, LLC (Carlyle, IL, United States), and Acuity Ag Solutions, LLC (Carlyle, IL, United States). Loin depth (cLD), backfat (cBF), and hot carcass weight were measured on terminal three-way crossbred pigs. Birth weight and date as well as harvest date were recorded, and harvest age was calculated. Carcass average daily gain (**cADG**) was calculated for each individual by subtracting birth weight from hot carcass weight and dividing by harvest age.

All individuals were crossbred gilts and barrows, progeny of Duroc sires, and different purebred or crossbred dam lines. Animals were born in three sow farms and raised between September 2015 and November 2019 on two commercial grower–finisher flows (Bergamaschi et al., 2019). The initial data set was composed by 135,768 records, which were edited by removing outliers (exceeding three SD from the mean) for each trait. Table 1 shows the descriptive statistics for the traits of interest; a total of 126,052 records were available for statistical analyses.

**TABLE 1:** Descriptive statistics for the traits used in the study (*n* = 126,052).

**Trait^1^**	**Mean**	**SD^2^**
cBF, mm	18.7	4.11
cLD, mm	67.3	7.03
cADG, kg/d	0.54	0.12

*^1^cBF, carcass backfat depth; cLd, carcass loin depth; cADG, carcass average daily gain. ^2^SD, standard deviation.*

Piglets were moved to different nursing/finishing facilities at weaning (18.7 ± 4.11 days). Individuals were considered ready for harvest at a target weight of approximately 136 kg. Harvest occurred on average at 178 days ± 10.6 days of age.

During the grow–finish period, a standard pelleted gender-specific dietary program was used. Individuals were monitored daily and received standard vaccination and emergency medication. For details on diet composition, vaccination, and medication during nursery, growth, and finish periods, see Lu et al. (2018).

For the data analyses, all animals were allocated into 57 contemporary groups (**CG**) based on the combination of farm month–year of birth. Frequency in each cell ranged from 153 to 6,025 individuals. Individuals were also allocated into 84 slaughter batches (batch) based on the concatenation of finisher farm and harvest date. Carcass quality traits were measured 24 h postmortem using a Fat-O-Meter system (Frontmatec A/S, Kolding, Denmark) at approximately the 10th rib. The pedigree file animals traced back nine generations, including a total of 2,248 animals.

Phenotyped individuals were progeny of 407 sires, and 279 of them were genotyped using the Illumina porcineSNP60 BeadChip (Illumina, Inc., San Diego, CA, United States). Crossbred individuals were born in a total of 20,525 litters; each sire was parent to 1–546 litters.

### Weather Data

Meteorological data were extracted from the National Climatic Data Center Quality Controlled Local Climatological Data^1^ database at the National Oceanic and Atmospheric Administration and consisted of hourly values of air temperature (°C) and relative humidity (%) measured during the period between 2015 and 2019. Records were extracted from three different weather stations (*Springfield*, *Quincy*, and *Lawrenceville*) distributed in the state of Illinois (IL, United States) and closest to the grower–finisher facilities. Weather stations were assigned to farms based on their zip code using the “zipcode” (Breen, 2012) and “geosphere” (Hijmans, 2019) packages of the R software (R Core Team, 2016).

The hourly temperature–humidity index was calculated using the formula proposed by Zumbach et al. (2008) as follows:

(1) THI=T-(0.55-0.0055×RH)⁢×⁢(T-14.5)

where T is the observed temperature in degree Celsius (°C) and RH is the observed relative humidity on a 0–100 scale. Average daily values for each climatic variable were then calculated.

### Statistical Analysis

In order to better investigate the patterns of thermal stress, three lifetime periods were defined. Using the birth date of the animal, thermal load was defined for three time intervals (60–92 days, 92–122 days, and 122–152 days) of age for each individual in the study. The average daily temperature, relative humidity, and temperature–humidity index for the daily values of each time interval were calculated. The nine resulting environmental covariates (three time periods by three climate variables) were then merged to each individual’s phenotypic record. For each of the 27 trait-by-environmental-covariate combination, linear models were used in order to evaluate the response of a considered trait to a specific environmental covariate. The first linear model was chosen, instead of Pearson correlation, for the possibility to adjust for other systematic effects. The model specifications for the first model were.

(1) $$y_{i\,j\,k\,l\,m}=\alpha \,1+C\,F_{i}+P\,a\,r_{j}+G_{k}+D_{l}+\beta_{\varphi_{1\,m}}+\varepsilon_{i\,j\,k\,l\,m\,n}$$

where y_*ijklm*_ is the phenotypic measure for one of the three traits (cBF; cLD; cADG), α is the intercept, *CF*_*i*_ is the effect of cross-fostering (*I* = 0 or 1), *Par*_*j*_ is the effect of the dam parity (*j* = 1–8), *G_k* is the effect of the gender of the individual (*k* = gilt or barrow), *D_l* is the effect of the dam genetic line (*l* = 1– 22), β is the fixed regression coefficient on the environmental covariate, *φ*_1m_ is the environmental covariate vector (expressed as first-order Legendre polynomial) at value *m*, and ε_*ijklmn*_ is the residual. In a second step, a first-order random regression model (RRM) was implemented using the *MCMCglmm* package of R (Hadfield, 2010) with the following specifications:

$$y_{i\,j\,k\,l\,m\,n\,o\,p\,q}=\alpha \,1+C\,F_{i}+P\,a\,r_{j}+G_{k}+D_{l}+\beta_{\varphi_{1\,m}}$$

(2) $$+a_{0\,n}\,1+a_{1\,n}\,\varphi_{1\,m}+b_{o}+l_{p}+\varepsilon_{i\,j\,k\,l\,m\,n\,o\,p\,q}$$

where *y*_*ijklmnopq*_ is the phenotypic measure for one of the three traits (cBF; cLD; cADG); α, *CF*_*i*_, *Par*_*j*_, *G_k*, and *D*_*l*_are as in Equation 2; φ_1*m*_ is the chosen environmental covariate (Table 2); β is the fixed regression coefficient on the environmental covariate at the population level; *a*_*0n*_ and *a*_*1n*_ are random regression coefficients for the additive genetic effect of sire *n* for the intercept and slope terms, respectively; *b_o* is the random permanent environmental effect of harvest batch (84 levels); *l_p* is the random permanent environmental effect of birth litter (20,252 levels); and ε_*ijklmnopq*_ is the random residual.

**TABLE 2:** Coefficients of determination and Bayesian information criterion values for the models assessing the impact of different heat load functions on the traits of interest.

**Environmental measure**	**Time period**	***R*^2^**	**BIC, model1**	**BIC, model2**
**Carcass average daily gain**				
THI	60–92, d	0.041	−241192.87	−241161.44
Temp	60–92, d	0.040	−241186.62	−241157.38
RH	60–92, d	0.041	−241672.4	−241649.27
THI	92–122, d	0.055	−241775.51	−241746
Temp	92–122, d	0.055	−242126.2	−242105.38
RH	92–122, d	0.048	−241340.31	−241352.62
THI	122–152, d	0.041	−242626.65	−242607.3
Temp	122–152, d	**0.057**	−242805.43	−242773.22
RH	122–152, d	0.041	−**241016.71**	−**240975.52**
**Carcass backfat depth**				
THI	60–92, d	0.135	640065.622	N.C.
Temp	60–92, d	0.134	640037.831	N.C.
RH	60–92, d	0.139	639893.692	N.C.
THI	92–122, d	0.125	640068.172	N.C.
Temp	92–122, d	0.125	640140.87	N.C.
RH	92–122, d	**0.144**	**639767.656**	N.C.
THI	122–152, d	0.135	639935.033	N.C.
Temp	122–152, d	0.136	N.C.	N.C.
RH	122–152, d	0.125	N.C.	N.C.
**Carcass loin depth**				
THI	60–92, d	0.014	N.C.	N.C.
Temp	60–92, d	0.015	N.C.	N.C.
RH	60–92, d	0.011	N.C.	N.C.
THI	92–122, d	0.022	814767.925	N.C.
Temp	92–122, d	0.022	814781.392	N.C.
RH	92–122, d	0.017	814757.367	814776.72
THI	122–152, d	0.014	814725.541	N.C.
Temp	122–152, d	**0.026**	**814701.658**	N.C.
RH	122–152, d	0.011	814797.207	N.C.

*BIC, Bayesian information criterion; Model 1 is the first-order Legendre polynomial random regression model; Model 2 is the second-order Legendre polynomial random regression model; Temp, daily average temperature (°C); RH, daily average relative humidity (%); THI, daily average values of temperature – humidity index (°C); N.C., not converged. The values in bold indicate the largest coefficient of determination or smallest Bayesian information criterion value obtained for each trait.*

The vectors for the sire effects were assumed as

$$\left[\begin{array}{c} {\text{a}}_{0}\\ {\text{a}}_{1} \end{array}\right]∼N\,\left(0,\text{H}⊗\text{G}\right)$$

where G is a 2 × 2 (co)variance matrix for the intercept and slope effects, respectively:

$$G=\left[\begin{array}{cc} \sigma_{0}^{2} & \sigma_{01}\\ \sigma_{10} & \sigma_{1}^{2} \end{array}\right]$$

where $\sigma_{0}^{2}$ is the sire variance for the intercept term, $\sigma_{1}^{2}$ is the sire variance for the slope term, and σ_10_ and σ_01_ are the covariance between the two aforementioned effects. The **H** matrix was constructed using the preGSf90 software (Aguilar et al., 2014; Misztal et al., 2014) blending the pedigree and SNP-derived genomic relationship matrices (Legarra et al., 2009). The harvest batch and litter permanent environmental effects were assumed as uncorrelated random effects with a mean equal to 0 and variance equal to the estimated variances $\sigma_{b\,a}^{2}$ and $\sigma_{l\,i}^{2}$, respectively. Residuals were considered being allocated in ten classes each with a different estimated residual variance $\sigma_{e\,t}^{2}$, where *t* is the class number. The classes were defined based on the nine deciles of the environmental covariate. Using this criterion, records are classified minimizing differences in the environmental covariate, and all classes have (approximately) the same number of records. A second-order Legendre polynomial model was also implemented, which included an additional fixed regression term and one additional random regression term for the additive genetic effect.

A total of 300,000 Gibbs samples were generated, while discarding the first 50,000 as burn-in and thinning every 50 samples. Posterior means and standard deviations of the remaining 5,000 samples were used as estimates and standard error for the (co)variance components. The goodness of fit was measured by the coefficient of determination (R^2^) for the model in 1 and by the Bayesian information criterion for the model in 2 (both for the first-order and second-order polynomials). Results are reported in Table 2.

### Estimation of Heritability Across the Range of the Environmental Covariate

The additive genetic (co)variance structures of individual sire across the range of the environmental covariate (**Γ**) was defined as

Γ=Φ⁢’⁢G⁢Φ

where G is the estimated (co)variance matrix between the intercept and slope terms and Φ is a matrix containing a column of “1” (intercept) and the environmental covariate. Heritability at each single value *m* of ENV ($h_{m}^{2}$) was calculated as

$${\text{h}}_{\text{m}}^{2}=\frac{\Gamma_{\text{mm}}}{\Gamma_{\text{mm}}+\sigma_{\text{ba}}^{2}+\sigma_{\text{li}}^{2}+\sigma_{\text{et}}^{2}}$$

where Γ_mm_ is the *m*^*th*^ value of the diagonal of **Γ**, and $\sigma_{b\,a}^{2}$, $\sigma_{l\,i}^{2}$, and $\sigma_{e\,t}^{2}$ are as defined above. Phenotypic predicted values at the population were calculated as *r*_*m*_=α+β*φ*_1m_, while phenotypic predicted values at the sire level (i.e., reaction norms) were calculated as *r*_*mn*_=(α+α_0*n*_)×1(β+*a*_1*n*_)×*φ*_1*m*_ where *a_0* and *a_1* were as in model 2, *r_m* is the prediction at value *m* at the population level, and *r*_*lm*_ is the prediction at value *m* for individual *n*. Genotyped sires were ranked based on their genomic estimated breeding values for the intercept *a_0* and slope *a_1* in terms of the first-order random regression model. Twenty genotyped sires showing *a_0* were labeled as **intHi** while those showing lowest values were labeled **intLo**. Similarly, the twenty genotyped sires showing the highest *a_1* were labeled as **sloHi** and the sires showing the lowest values were labeled as **sloLo**.

## Results

### Characterization of Climatic Conditions

**FIGURE 1:**
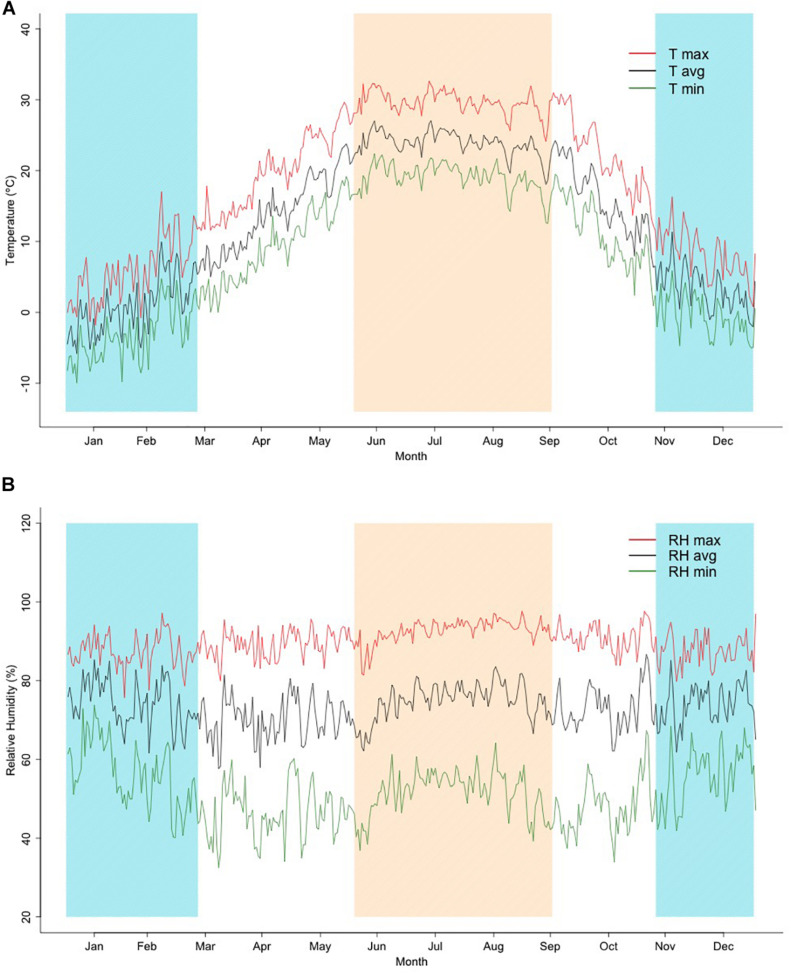
**(A,B)**. Average values of daily maximum (T max), average (T avg), and minimum (T min) temperatures, and daily maximum (RH max), average (RH avg), and minimum (RH min) relative humidity through the year for years 2015–2019 as used in this study. Shadowed regions represent hot (top-left to bottom-right lines) and cold (bottom-left to top-right lines) seasons.

The summary of monthly mean temperatures and humidity is illustrated in Figure 1, which shows the trend of average, minimum, and maximum temperature as an average of each day over the studied period. Three different periods can be observed: a cold season, including the days with an average daily temperature below 10°C, a mild season with days showing between 10 and 20°C, and a hot season, which included the days with temperature over 20°C. The coldest days of winter occurred in December and January, which were associated with a mean daily maximum and minimum temperatures around 3.2 and −5.3°C, respectively. Intermediate temperature values were found for the months of April, May, and October, in contrast with the extreme minimum temperatures that occurred during the period from January to February (values varied between −10.2 °C and −8.5°C). Temperature reached the maximum value of 32.3°C in July. Relative humidity was constant over the year with the lower minimum values observed during the spring season, ranging between 69.6% in April and 70.8% in May. A larger variation within season was observed for relative humidity. The pattern shows a large range within the cold months (57.6%) and the hot period (49.6 %). For the temperature–humidity index, the trends observed were very close to those estimated for corresponding temperatures, with low values in winter and high during the summer (results not shown).

As reported in Table 2, all coefficients of determination values were small, indicating that only a small part of phenotypic variation is explained by the weather variables and systematic effects. *R*^2^ and BIC were mostly consistent in pointing at the best fitting models, with the exception of cADG.

For cBF, the best predictor (*R*^2^ = 0.144, BIC = 639767.66) was RH recorded in the period between 92 and 122 days of age. For this trait, none of the second-order RRM models reached convergence. The best fit for cLD occurred using Temp recorded during the 122–152-d period (*R*^2^ = 0.026, BIC 814701.66). Again, just one second-order RRM model reached convergence. Conversely, there was no concordance for the best environmental covariate for cADG, since the largest R^2^ was generated by Temp recorded during the 122–152-day period (*R*^2^ = 0.057) while the lowest BIC was generated by RH recorded in the period between 122 and 152 days (BIC equal to −241016.71 for the first-order RRM, −240,975.52 for the second-order RRM).

### Genetic Parameters for Estimated Traits

**FIGURE 2:**
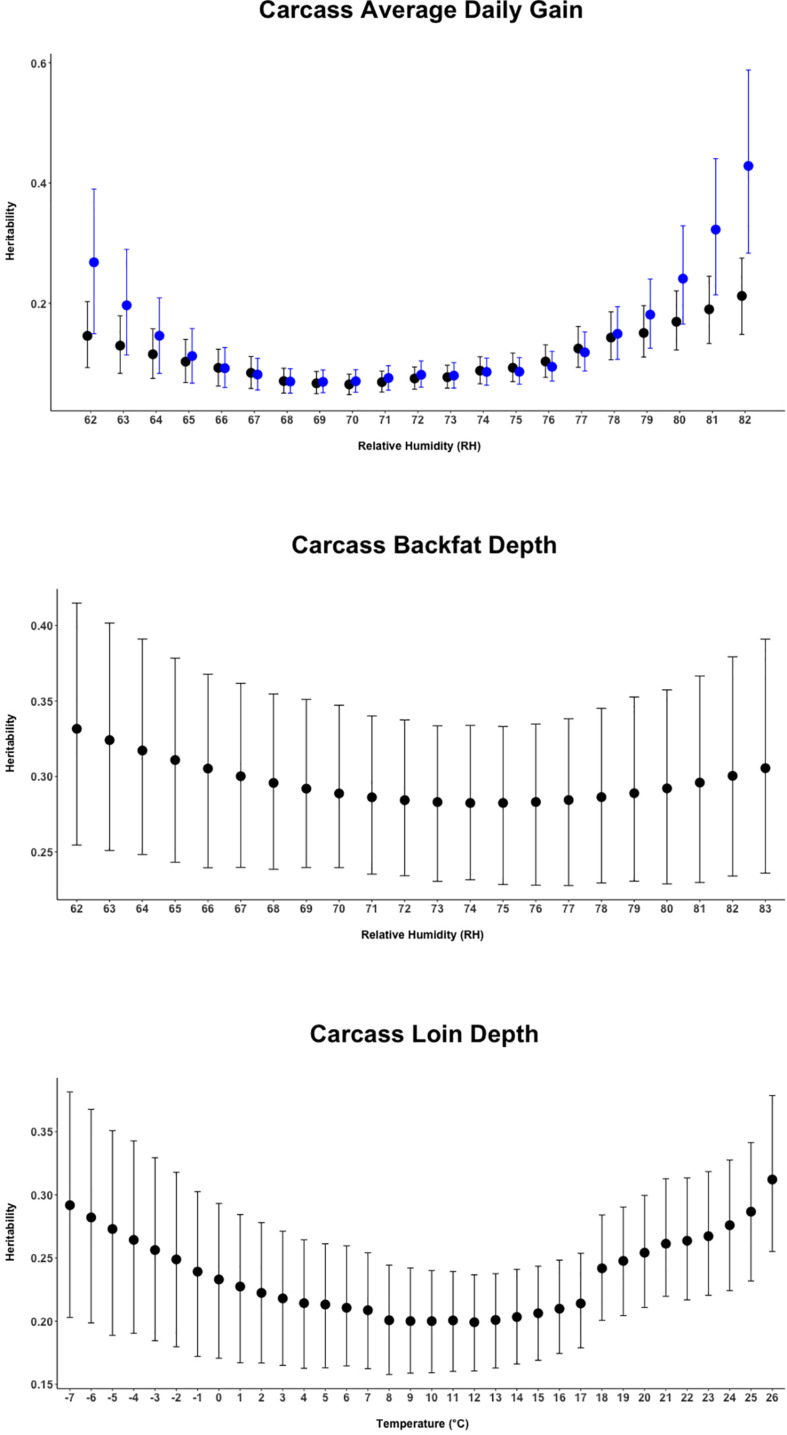
Heritability estimates (95% empirical confidence intervals) for the three carcass quality traits of animals over the range of the respective climatic variable. The black dots report the estimates from the first-order Legendre polynomial random regression model. The blue dots report the estimates from the second-order Legendre polynomial random regression model (available for Carcass Average Daily Gain only).

Random regression models were used to evaluate the effect of heat stress and potential genetic control of heat tolerance for carcass quality traits of crossbred pigs. Heritability estimates from the current study indicates a possible genetic improvement for heat tolerance by selecting for the direct genetic component of carcass quality traits under heat-stressed conditions. Results illustrated in Figure 2 show that cBF was moderately (∼0.32) heritable across all values of relative humidity, and a moderate heritability (∼0.30) for cLD was also found, with higher values at lower (below 0°C) and higher temperatures (above 18°C). For cADG, heritability estimates appeared to be larger at the extreme values of the environmental covariate, a pattern that was exacerbated when using the second-order polynomial.

**FIGURE 3:**
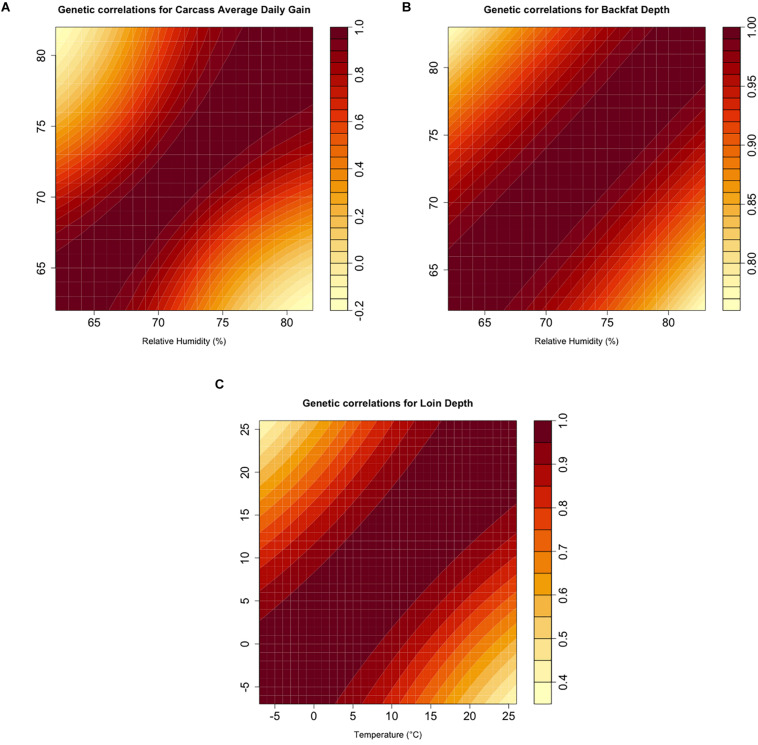
**(A–C)**. Estimates of additive genetic correlations using the genomic random regression model (2) for the corresponding climatic variable and carcass quality trait combinations.

Genetic correlations are summarized as a heat map for each analyzed trait in Figure 3. All traits showed a non-unity genetic correlation between extreme values of the environmental covariate. Correlations for cADG showed a value of 0.204 between the values of relative humidity corresponding to the 5th and 95th percentiles (i.e., 66 and 80%). cLD reached a value of 0.5 between the values corresponding to the values of Temp at the 5th and 95th percentiles (i.e., −4 and 25°C). Likewise, genetic correlations for cBF were the strongest, with 0.872 between the relative humidity 5th and 95th percentile values (i.e., 65 and 80%).

### Estimated Breeding Values From RRM

While fit measures for cADG suggested the use of the second-order RRM, we noted poor convergence for the sire solutions for the quadratic term even if fixing the variance components. Therefore, we decide to use the first-order polynomial model for this trait.

**FIGURE 4:**
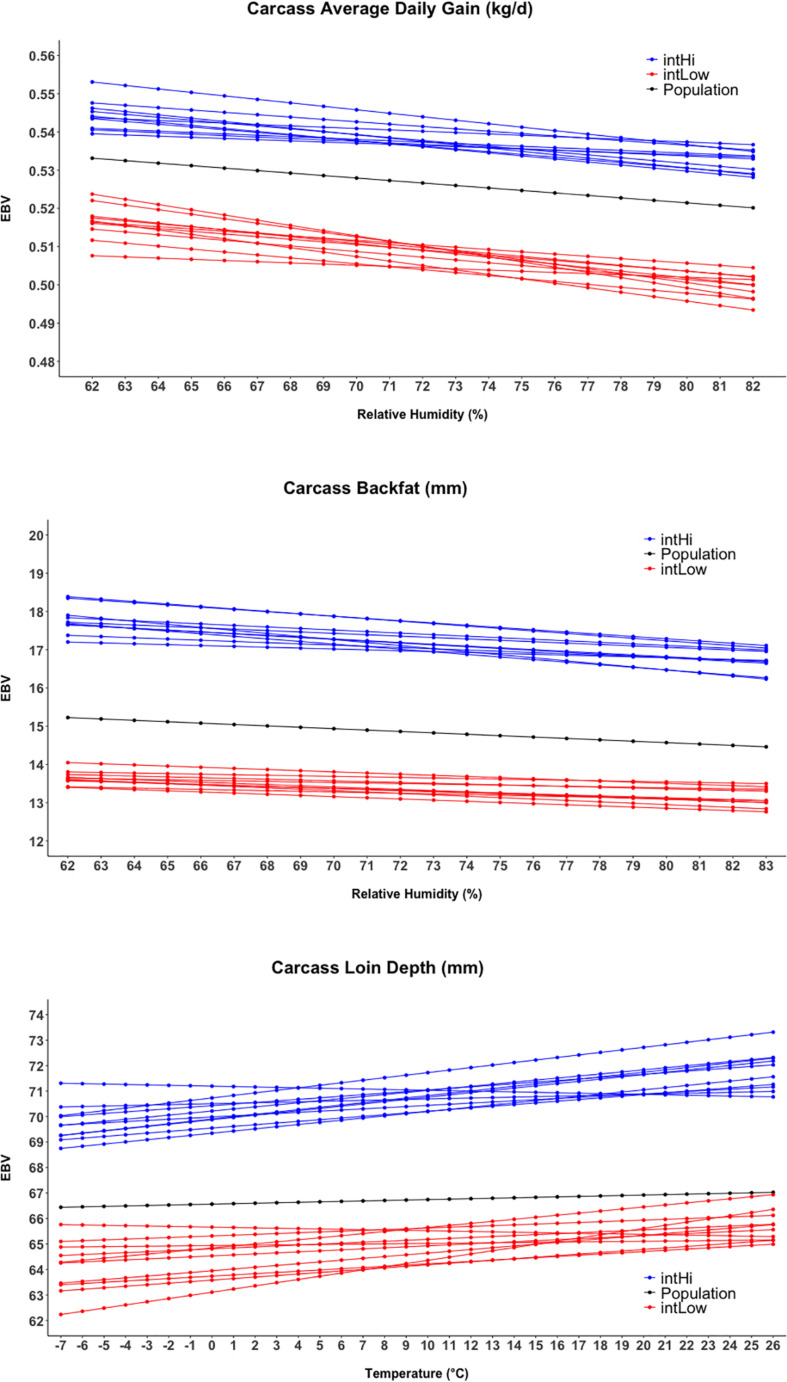
Reaction norms for the twenty sires showing the higher and lower estimated breeding values (EBV) for the intercept term of the random regression model.

**FIGURE 5:**
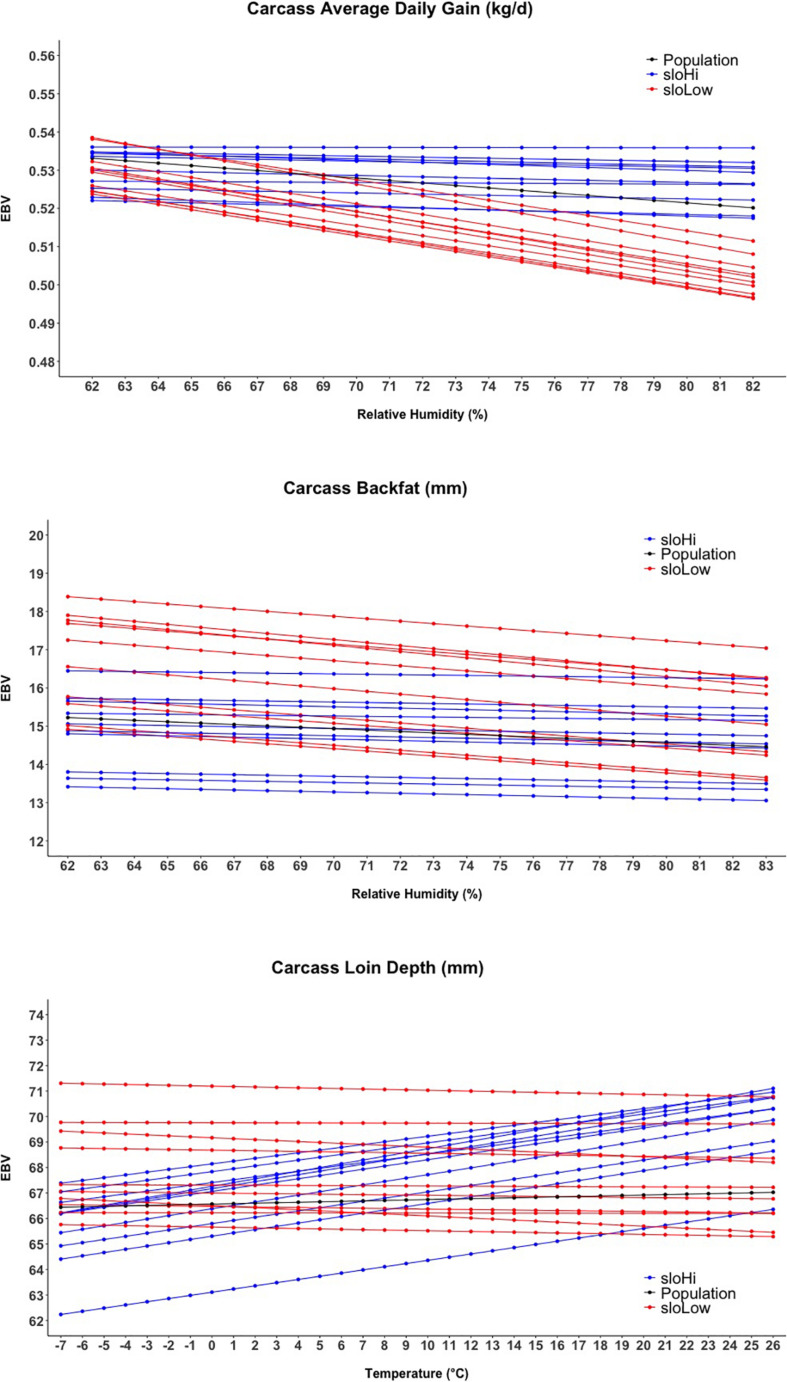
Reaction norms for the twenty sires showing the higher and lower estimated breeding values (EBV) for the slope term of the random regression model.

Figure 4 shows the reaction norms for intHi and intLow sires, together with the population reaction norm. Results show a large constant difference between the two groups for cBF, cLD, and cADG. Among all traits, the largest differences between sire groups were observed for cBF, where population-level estimates declined from 15.2 to 14.5 mm and the difference between the two groups was about 40 mm. Considering cADG, heat (humidity) stress caused the trait to decline from 0.533 to 0.520 kg/days at highest humidity; the difference between intHI and intLo for this trait was of 0.031 kg/days at intermediate humidity values (70–75%). An increase with Temp was shown for intHi in cLD, though the population-level increase in cLD was less than 1 mm. For cLD, the difference between the two groups was about 6 mm. For all traits, the difference between the intHi and intLo sire groups was larger than the average loss due to heat stress at the population level, and the two groups did not show evident differences in tolerance to heat stress. Figure 5 shows the sloHi and sloLo groups. No differences are found between the two groups in their performance across conditions, but sires show a large variation in their reaction norm slopes. Traits cBF and cADG report the sloHi sires (the most tolerant) showing flat reaction norms, which proves strong tolerance to heat stress, while sloLo sires show reaction norms with a drop in performance remarkably stronger than the population average. Trait cLD also shows large variability in their reaction norms. Here, sloLo sires show flat norms for cLD while sloHi sires show increasing reaction norms.

## Discussion

The phenotypic data used in this study came from a commercial system where three-way crossbred pigs are generated using single-sire semen, thus allowing the estimation of genetic parameters in a commercial environment. The growing–finishing units run on a fixed-weight system, where individuals are harvested when their body weight reaches the desirable value. However, harvest is not performed on an individual but on a batch-based basis, which allows some intra-batch variability in body weight and carcass composition. Descriptive statistics reported in Table 1 show that some (limited) variability in cADG exists (0.12 kg/day). The variability for this trait could be attributable to slower growth of individuals that are subject to heat stress, but also to differences in genetic background and diet fed to the different groups. The optimal market weight is generally set around 130 kg, though it can generally vary according to market needs.

### Genetic Parameters

The heritability estimates from the current study indicate the potential to perform selection by selecting for the direct genetic component of carcass quality traits both under comfortable and heat-stressed conditions (Figure 2), with heritability being non-null also under comfortable conditions. Generally, previous studies reported carcass weight as a moderately to highly heritable trait (Fragomeni et al., 2016a,b), but Zumbach et al. (2008) found low to moderate heritability of 0.14 and 0.28 for carcass weight in thermo-neutral and heat-stress conditions, respectively, being therefore larger under heat-stress conditions. A moderate heritability for carcass weight during heat stress was also indicated from Bradford et al. (2016) that, in a study conducted on beef cattle, reported values between 0.24 for weaning weight and 0.32 for yearly weight. Figure 2 shows heritability for cBF being constant (∼0.32) but lower than the heritability of 0.43 estimated by Ciobanu et al. (2011). Similar to those observed for cBF, the estimates reported for cLD (0.19–0.31) were lower than the estimates of 0.47 from many previous studies reported by Stewart et al. (1991) and Miar et al. (2014) above 18 and 19°C degrees of temperature. As previously proposed in other studies in dairy and beef cattle, the application of a random regression model allows also the estimation of genetic (co)variance components and breeding values over the whole trajectory of environment-dependent variables and that can be considered as an estimate of the magnitude of GxE (Mulder and Bijma, 2007). In this study, the trait that showed the strongest GxE magnitude was cADG followed by cLD. With 0.70 being the threshold suggested to declare the existence of GxE (Mulder and Bijma, 2007), it can be inferred that GxE was only detected for cLD and cADG.

Although RRMs have been previously used to model animal weight (live weight) in dairy and beef cattle (Meyer, 2000; Coffey et al., 2002; Bohlouli et al., 2013; Bradford et al., 2016), the use of this approach to assess the effect of environmental conditions on animal conformation or carcass quality trait is scarce in pigs. All available studies on the genetic component for heat tolerance in swine used the RRM to determine the genetic parameters as a function of heat load on growth traits or carcass weight (Zumbach et al., 2008; Fragomeni et al., 2016a,b).

### Reaction Norms and Identification of Thermo-Tolerant Genetic Material

The impact of heat stress found in this study was considerable for cADG and cBF. Lower carcass fatness at slaughter connected to the decline in feed intake is generally reported in heat-stressed pigs (Le Dividich et al., 1998). Thus, according with Le Bellego et al. (2002) and Renaudeau et al. (2011), the decrease in growth rate associated with thermal stress could be primarily a result of a decline in feed intake. As a consequence, a slight increase also in feed conversion ratio at a very high-temperature level is expected. During heat stress, feed intake is decreased in order to reduce the heat production associated with the digestion and metabolism of nutrient (Ross et al., 2015).

Similar to feed intake, cADG shows a decreasing response during the thermal load and is affected by the animal’s body weight with heavier pigs more susceptible to heat stress than lighter ones (Renaudeau et al., 2011). In our study, the animals belonging to the sloLow group for cADG reduced their performance from 0.53 to 0.51 passing from relative humidity of 62–82%. This sensitivity to warmer temperature supports the hypothesis for which during humid periods heat cannot be dissipated and pigs did not consume a sufficient amount of feed for normal gain.

The decrease in feed intake does not explain the weak loss in cLD when heat stress occurs. For this trait, the impact was approximately null and some families actually showed an increase in cLD when conditions involved heat stress. The increase in cLD was of 3 mm of cLD passing from −7 to 26°C of Temp, in contrast with the common observation on heat-stressed finishing pigs (Cruzen et al., 2015; Ross et al., 2015). This could be due to the fact that individuals are harvested when reaching market conditions, which is based on the assessment of muscle deposition. Individuals from the genetic families that show an increase in cLD under heat stress could show better muscle deposition than the average, therefore reaching market requirements at a lower body (and carcass) weight. In the present study, we attempted to define another trait, expressed as the ratio between cLD and hot carcass weight, as an indicator of muscularity (results not included). This could have validated the aforementioned hypothesis, but poor convergence of the models for this trait did not allow us to include it in the manuscript. Further research is needed to investigate the potential GxE for muscle deposition in pigs, in relationship to the heat stress impact on feed intake.

Differences in performance reported in this study between no thermal stress and maximum thermal stress scenario would have a large economic impact for producers in swine production, influencing carcass quality and consequently the profit function within the pork industry. The methodology proposed in this study using weather information to identify heat-tolerant animals could be a useful tool to improve the production system and implement the selection programs. Ideally, the use of this approach represents a breeding strategy to improve heat tolerance in relation to the farm resources.

The use of weather station climatic data was again proven valuable for a first estimation of reaction norm and heat tolerance of the different families. Outdoor records are a poor prediction of indoor condition, which does not allow the exact definition of comfortable and uncomfortable conditions. Further studies will need to consider indoor-recorded environmental data.

Farming systems could benefit more from including heat tolerance in the breeding programs of individuals that are resistant to extreme conditions. On the other hand, we found a partial antagonism between heat tolerance and productivity. Comparing the intHi and intLow sires for the three traits, we do not observe a strong difference in the slope of the reaction norms. Comparing the sloHi and sloLow sires, their performance under comfortable conditions appears to be different. For cBF, the most resilient individuals have a lower performance under comfortable conditions and approximately the same performance under heat stress. This could be related to their efficiency in converting feed into body weight instead of body heat. However, this hypothesis will need further studies to be proved.

If selection for increased resilience is performed, there will probably be a loss in performance under optimal conditions. This suggests that overall performance and tolerance to heat stress should be combined in an economic selection index, with different weights depending on the likelihood of certain conditions to occur in a particular system.

## Conclusion

A random regression model including genomic information was used to evaluate the effect of heat stress on carcass quality traits of crossbred pigs. Data used for this study came from commercial operations, making the presented results representative of the swine industry.

Performance under heat stress seems to be less or equally heritable than under comfortable conditions, but genetic variation still exists even under heat stress, indicating that the identification and selection of the most resistant animals is possible in order to implement the selection programs. A graphical analysis of the reaction norms shows that genetic material with improved heat tolerance is easily identifiable. The use of outdoor-recorded environmental measures can be valuable for early studies on the subject, but indoor-recorded measures will be needed for further studies.

The three traits studied showed a different impact of heat stress and different magnitude of genotype by environment interaction. Because of this, it will be a task of the breeder to determine the stronger economic value of heat tolerance (vs overall performance) for each trait.

Further research is needed for the heat tolerance of swine to overcome the complexity of selection of heat-tolerant animals, due to a partial antagonism between heat tolerance and overall performance.

## Data Availability Statement

The datasets presented in this article are not readily available because the raw datasets are property of the swine breeding companies and this information is commercially sensitive. Requests to access the datasets should be directed to Clint Schwab.

## Ethics Statement

Ethical review and approval was not required for the animal study because the data used in this study came from animal raised under commercial facilities for pork production. Written informed consent for participation was not obtained from the owners because the animals were property of some of the coauthors.

## Author Contributions

MU, NM, and FT conceived and designed this study. MU and MB carried out the analyses. MU, FT, NM, CM, and MB interpreted and discussed the results. MU wrote the first draft of the manuscript. ClS, JF, and CaS supervised the data collection and provided inputs for the analyses of the data. All the authors reviewed and approved the final manuscript.

## Conflict of Interest

The study used data that were provided as in kind by The Maschhoffs LLC. ClS, CaS, and JF were employed by The Maschhoffs LLC or Acuity Ag Solutions LLC at the time of submission. The results are commercially of interest to the above-mentioned companies but this interest did not influence the results presented in this manuscript in any matter. The remaining authors declare that the research was conducted in the absence of any commercial or financial relationships that could be construed as a potential conflict of interest.

## Footnotes

1 https://www.ncdc.noaa.gov/qclcd/QCLCD?prior=N
